# Analysis of tribological behaviors and regulating functions at elevated temperatures of microchannel interfaces prepared in Ti-base alloys

**DOI:** 10.1039/d3ra01986a

**Published:** 2023-05-24

**Authors:** Taiping Zhang, Na Xiao, Kang Yang, Feizhi Zhang, Yongxing Hao, Chenhua Zhang, Xue Yin, Yanfang Zhu

**Affiliations:** a School of Materials Science and Engineering, North China University of Water Resources and Electric Power Zhengzhou Henan 450045 China haoyongxing@ncwu.edu.cn; b Engineering Department, Huanghe University of Science and Technology Zhengzhou 450000 China 250801510@qq.com +86-372-2986271 +86-372-2986271; c Department of Mechanical Engineering, Anyang Institute of Technology Avenue West of Yellow River Anyang 455000 China; d School of Mechanical Engineering, Sichuan University of Science & Engineering 180# Xueyuan Street, Huixing Road Zigong 643000 China; e College of Marxism, Anyang Institute of Technology Avenue West of Yellow River Anyang 455000 China

## Abstract

For improving the tribological behaviors of traditional Ti alloys, high-nickel Ti alloy with sinusoidal micropores was prepared by laser additive manufacturing (LAM). MgAl (MA), MA-graphite (MA-GRa), MA-graphenes (MA-GNs), and MA-carbon nanotubes (MA-CNTs) were respectively filled into the Ti-alloy micropores to prepare interface microchannels through high-temperature infiltration. In a ball-on-disk tribopair system, the tribological and regulating behaviors of the microchannels in Ti-base composites were elucidated. The results showed that the regulation functions of MA were noticeably improved at 420 °C, resulting in their superior tribological behaviors than those at other temperatures. It could be concluded that GRa, GNs, and CNTs combined with MA further enhanced the regulation behaviors compared to individual MA lubrication. The following regulation factors were responsible for the excellent tribological properties: the interlayer separation of graphite, which accelerated the plastic flow of MA, improved the interface crack self-healing of Ti-MA-GRa, and regulated the friction and wear resistance abilities. Compared with GRa, GNs were easier to slide, and helped to produce a greater deformation of MA, facilitating a good self-healing of cracks, and further enhancing the wear regulation of Ti-MA-GNs. CNTs showed good synergism with MA to allow the rolling friction, which effectively repaired the cracks to improve interface self-healing, resulting in a better tribological performance of Ti-MA-CNTs compared to Ti-MA-GRa and Ti-MA-GNs.

## Abstract 2

This work reports tribological behaviors and self-regulating functions of microchannel interfaces prepared in high-nickel Ti alloys, which is useful for enhancing service life and the usage accuracy of Ti-base aero/avia-components.

## Graphical abstract

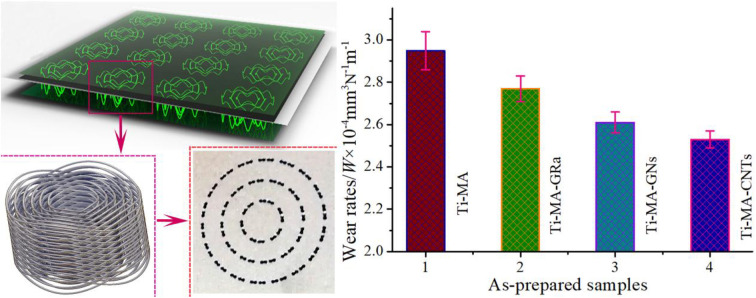

## 1. Introduction

In recent years, lightweight Ti-based components have been abundantly used in aerospace and biomedicine industries, as well as in other important applications that use their tribological properties.^1,2^ Ti alloys have attracted significant interest due to their confirmed friction and wear behaviors, especially under extreme conditions, which are crucial for increasing the service life and operation accuracy of lightweight components.^3^ However, the conventional Ti–6Al–4 V used for many components has shown poor friction and wear behaviors and has become the key obstacle to increasing the service life of components and devices. Therefore, further improvements in the friction and wear behaviors of Ti alloys are required. Available methods to prepare self-lubricating composites include the use of bulk self-lubricating samples,^4^ composite coatings,^5^ and poriferous materials.^6^

For improving the tribological behaviors of Ti-based bulk samples, SnAgCu and Al_2_O_3_ lubricants have been successfully filled into Ti-based composites,^7^ and showed that the SnAgCu and Al_2_O_3_ lubricants were helpful for improving the wear resistance. SnAgCu and Al_2_O_3_ were added into high-nickel Ti-base alloys, and their sliding wear behaviors were examined.^8^ The results showed that during wear, the Al_2_O_3_–SnAgCu films exhibited plastic deformation for low strengths of SnAgCu, which reduced the binding ability of Al_2_O_3_ with frictional surfaces, thus accelerating Al_2_O_3_ rotation and acquiring a friction coefficient of almost 0.20 and a wear rate of approximately 3.39 × 10^−4^ mm^3^ N^−1^ m^−1^. However, the solid lubricants SnAgCu and Al_2_O_3_ bonded strongly with the Ti-base bulk materials, and their dense organizations restricted the migration of lubricants. This led to only a slight enrichment in the friction interface, thereby imposing restrictions on the unlimited reductions in the friction coefficients and wear rates.

For good enhancements of the tribological behaviors of Ti-matrix samples, a hard lubricating clad layer was suggested for preparation on a Ti-alloy substrate using a pre-placed powder cladding technique.^9^ The results showed a rigorous decrease in the friction coefficient, which was 14.5 times much better than that of the Ti-substrate. For achieving a significant enhancement of the tribological behaviors, Ti-matrix composite coatings modified with Y_2_O_3_ were successfully fabricated on a Ti-alloy base by laser cladding. The results indicated that the superior tribological properties of coatings could be mainly attributed to the increased load-bearing provided by the large-sized TiN and TiB units, while the increased load-transferring strength mainly resulted from the fine TiB whiskers. A surface coating fabricated with 1 wt% Y_2_O_3_ showed the lowest wear loss, and the main wear mechanism was abrasive wear.^10^ However, significant differences in thermal expansion were found between the coatings and corresponding substrates, which led to the coatings being desquamated, and ultimately lubrication failure, thus restraining the tribological applications of the coatings.

With an increased focus on laser additive manufacturing (LAM), poriferous materials initially attracted much attention for utilization in applications associated with friction, wear, and lubrication. For accelerating Al_2_O_3_ and SnAgCu migration and reducing the desquamation of the coatings from their substrates, a microchannel filled with SnAgCu–Al_2_O_3_ was prepared into friction surfaces of TC_4_-base composite using LAM and high-temperature penetration.^11^ During wear, under the guidance of the microchannels, SnAgCu and Al_2_O_3_ migration was noticeably accelerated, leading to SnAgCu and Al_2_O_3_ accumulation on the wear interface, thus facilitating an excellent lubrication. Hence, LAM was validated as a successful preparation process for poriferous materials, thus providing an important reference for improvements in the tribological performances of Ti alloys.

The beneficial properties of MgAl (MA), such as a density of 1.5–1.8 g cm^−3^ and melting point of 420–453 °C, have been extensively utilized in various industries, including shipbuilding, aviation, and biomedicine.^12,13^ Adding MA into Ti alloys promotes their outstanding tribological behaviors at room temperature;^14^ in particular, the excellent collaboration of the MA with graphene and Al_2_O_3_ observably improved this aspect. Additionally, carbon nanomaterials (graphite (GRa), graphene (GNs), and carbon nanotubes (CNTs)) have been reported to be attractive lubricants for achieving a good lubricity of metal–matrix composites.^15–18^ This has greatly enhanced the tribological performances of carbon films and nanocomposite coatings. However, only a few studies have reported the associated usage of MA alloys with GRa, GNs, and CNTs, respectively, at elevated temperatures. In particular, the regulation functions of GRa, GNs, and CNTs on the tribological behaviors of MA-GRa, MA-GNs, and MA-CNTs have been rarely reported.

In gear-driven systems in aerospace, gears composed of Ti alloys have been applied to mesh with fixed Si_3_N_4_ internal gears to achieve excellent momentum transmission.^19^ Further, the cam mechanism has been tried with an aim to execute linear motion in aeronautical propulsion systems. During the propulsion, a sliding friction behavior of a Ti-alloy-based cam component relative to the spherical Si_3_N_4_ pushrod was observed, showing that the poor tribological performance constituted the main cause for the transmission failure of the Ti-alloy cam. To address this issue, new-style Ti-base alloys were prepared *via* LAM. Sinusoidal micropores in the frictional interfaces were respectively filled with MA, MA-GRa, MA-GNs, and MA-CNTs lubricants to prepare microchannels, for achieving improvements in the lubrication of the Ti alloys. The preparation process of the microchannel is elucidated in the following sections.

## 2. Experimental procedures

### 2.1. Material synthesis

#### 2.1.1. Synthesis of the micropores

**Fig. 1:**
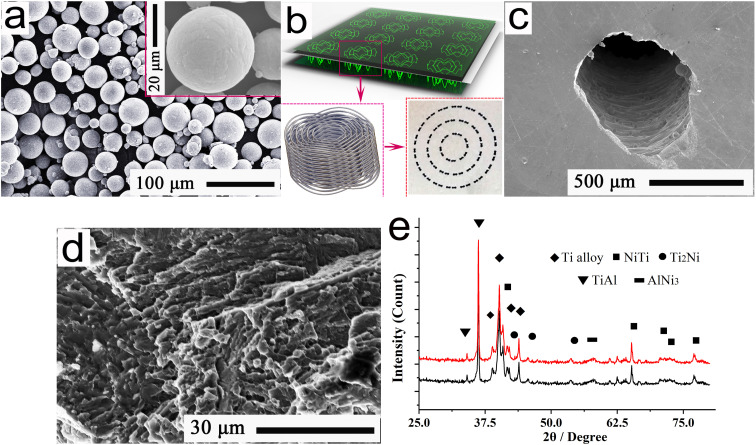
(a) Typical EPMA pattern of the spherical powders; (b) morphologies of the sinusoidal micropores; (c) FESEM image of the single micropore; (d) cross-section morphology; (e) XRD diffraction pattern of Ti alloy.

Commercial powders (purity: 99.5%; diameter: 20–40 μm) of Ni, Nb, Al, Zr, Si, Mo, Y, and Ti, in the nominal mass ratio of 16 : 7: 4 : 3: 0.55 : 0.35 : 0.25 : 68.85, were applied in this study. After being mixed in a vibration mill operated at frequency of 40–50 Hz, the mixed powders were loaded into a vacuum crucible of a gas-atomizer for preparing spherical Ti-alloy-base powders. At temperatures ranging from 1450–1500 °C, the melted Ti-alloys were incorporated into ultrapure water, leading to the formation of spherical powders under a fast-cooling environment. After detection and sifting for main diameters ranging from 40–75 μm, spherical powders were obtained. The EPMA (electroprobe microanalyzer) results of the powders can be seen in Fig. 1a.

As is well established,^14,20^ a large amount of microchannels, that consist of micropores and the lubricants, can result in the observed enhancements of tribological behaviors; but, these enhancements are obtained at the cost of a degraded bearing strength. Hence, with an important reference to ref. 14, the main parameters of the micropores were optimized using finite element simulation and friction measurements, finally demonstrating a channel diameter of 700 μm, sinusoidal amplitude of 1500 μm, sinusoidal periodic of 2π, channel interval of 3.5 mm, and poriferous thickness of 2 mm. In accordance with the applied models (see Fig. 1b) of sinusoidal micropores, a 3D metal printer of No. HBD-100D was used to incorporate the as-prepared spherical powders in the LAM Ti-alloy process. For the LAM, the following parameters were used to prepare the Ti-alloy-base samples with dimensions of 30 cm × 30 cm × 8 cm: a laser power of 130–150 W, layer thickness of 12–20 μm, width of 40 μm, velocity of 6000 mm s^−1^, oxygen content of 45–60 ppm, and an atmosphere of more than 98.5% argon.

After the LAM, the typical morphologies of the sinusoidal micropores could be observed in Fig. 1b and c. A cross-sectional morphology of the Ti alloy was well observed using field emission scanning electron microscopy (FESEM), and the result has been shown in Fig. 1d, ensuring the dense organization. Further, XRD diffraction pattern of Ti-base alloy is shown in Fig. 1e. As shown in this figure, the Ti-base alloy mainly consisted of Ti, NiTi, Ti_2_Ni, TiAl, and AlNi_3_ phases. This indicated that the sinusoidal micropores had been successfully prepared in the Ti-alloy-base interfaces.

#### 2.1.2. High-temperature infiltration of the microchannels

MA, MA-GRa, MA-GNs, and MA-CNTs were purchased from Nanjing XFNANO Materials Tech Co., Ltd, and were then filled into the micropores for preparing the microchannel interfaces of Ti-base alloys *via* high-temperature infiltration. GRa, GNs, and CNTs lubricants of almost 1.50 wt% mass fraction were chosen to shake and mix with the MA for the successful synthesis of the MA-GRa, MA-GNs, and MA-CNTs, respectively. Subsequently, MA, MA-GRa, MA-GNs, and the MA-CNTs were respectively filled into the sinusoidal micropores of Ti-alloy interfaces. After spark plasma sintering (SPS), the respective microchannels were prepared into the corresponding samples involving Ti-MA, Ti-MA-GRa, Ti-MA-GNs, and Ti-MA-CNTs. With the SPS instrument model no. D. R. Sinter®, SPS3.20, the following parameters were used in this study: infiltration heating rate of 30–45 °C min^−1^, temperature in the range of 450–550 °C, pressure of 32–35 MPa, and an infiltration time of 120–150 min.

**Fig. 2:**
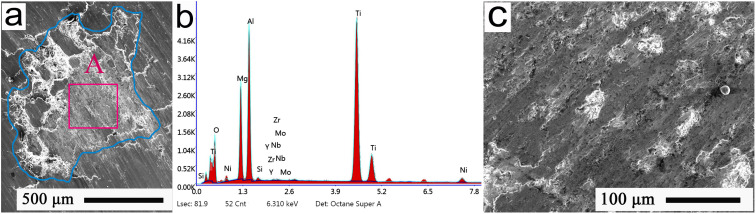
(a) Typical FESEM morphology of the single microchannel; (b) EDS spectrum of the material in (a), (c) amplified morphology of the region bounded by the red rectangle A in (a).

The MA-filled microchannel was obtained after high-temperature infiltration, and then was polished and cleaned and dried in the oven for 10 min, thus acquiring the dried samples. Subsequently, the representative FESEM morphology of the MA-filled interface is shown in Fig. 2a, and its EDS spectrum is given in Fig. 2b; further, an amplified morphology of the region in the red rectangle A can be observed in Fig. 2c. These verify that the MA had been well filled into the micropores to acquire the microchannels.

**Fig. 3:**
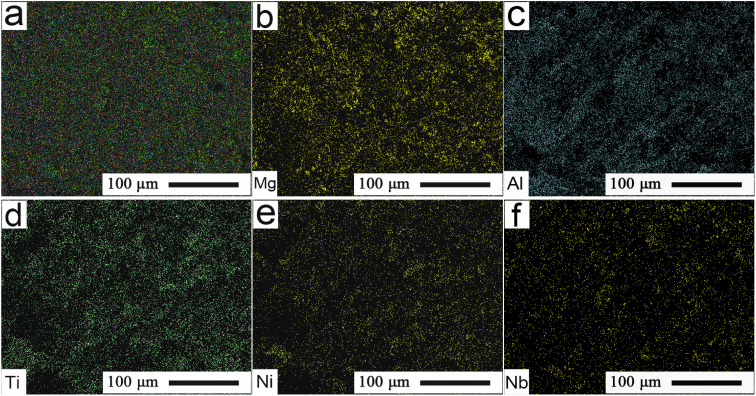
(a) Typical mapping pattern and (b)–(f) FESEM distributions of the primary elements of the interface microchannel presented in Fig. 2c.

Fig. 3 shows the mapping pattern and FESEM distributions of the primary elements presented in Fig. 2c. As shown in Fig. 3b and c, Mg and Al elements predominantly appeared in the white regions, and demonstrated good distributions in the microchannel. The typical distributions of Ti, Ni, and Nb elements of the Ti-alloy substrate could also be well observed in Fig. 3d–f. This was possibly because the substrate elements, such as Ti, Ni, and Nb, of the Ti alloys were towed to the microchannel surface during polishing. This ensured that the MA lubricants were well filled into the interface microchannel of the Ti alloy, and thus showed homogeneous distributions.

### 2.2. Friction and wear tests of the microchannel samples

**Fig. 4:**
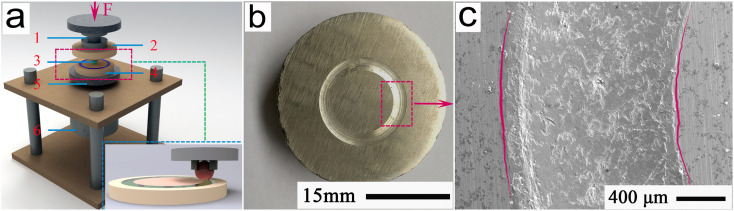
(a) Typical schematic of the ball-on-disk tribopair system; inset shows the frictional contact between the Si_3_N_4_ ball and the disk sample indicated by the dotted rectangle; (b) and (c) typical morphologies of the frictional interfaces.

In order to perform a good assessment of the friction and wear performances of the microchannel samples sliding against the fixed Si_3_N_4_ balls, the ball-on-disk tribopair system of model no. MFT-5000 was adopted in this study. Fig. 4a shows a typical schematic of a ball-on-disk tribopair system. The inset indicates the frictional contact between the Si_3_N_4_ ball and the microchannel sample, as exhibited by the dotted rectangle. Herein, the main components were named as the corresponding numbers: 1-force sensor; 2-upper holder; 3-Si_3_N_4_ matching ball; 4-LAM sample; 5-lower holder; and 6-stepping motor. As shown in Fig. 4a, in the ball-on-disk tribopair system, the lower holder with the microchannel sample was driven by a closed-loop stepping motor and rotated against the Si_3_N_4_ ball of 6 mm diameter and approximately 15.5 GPa hardness. Under applied loads of 12 N and 24 N, with service temperatures ranging from 0 °C to 560 °C, sliding tests for 80 min and 95 min of friction and wear were carried out at a relative humidity of 45–50%. Herein, the applied load of 24 N and sliding wear time of 95 min were adopted to evaluate the self-healing behavior of the wear interface, and to examine the self-regulating function of the friction and wear behavior.

**Fig. 5:**
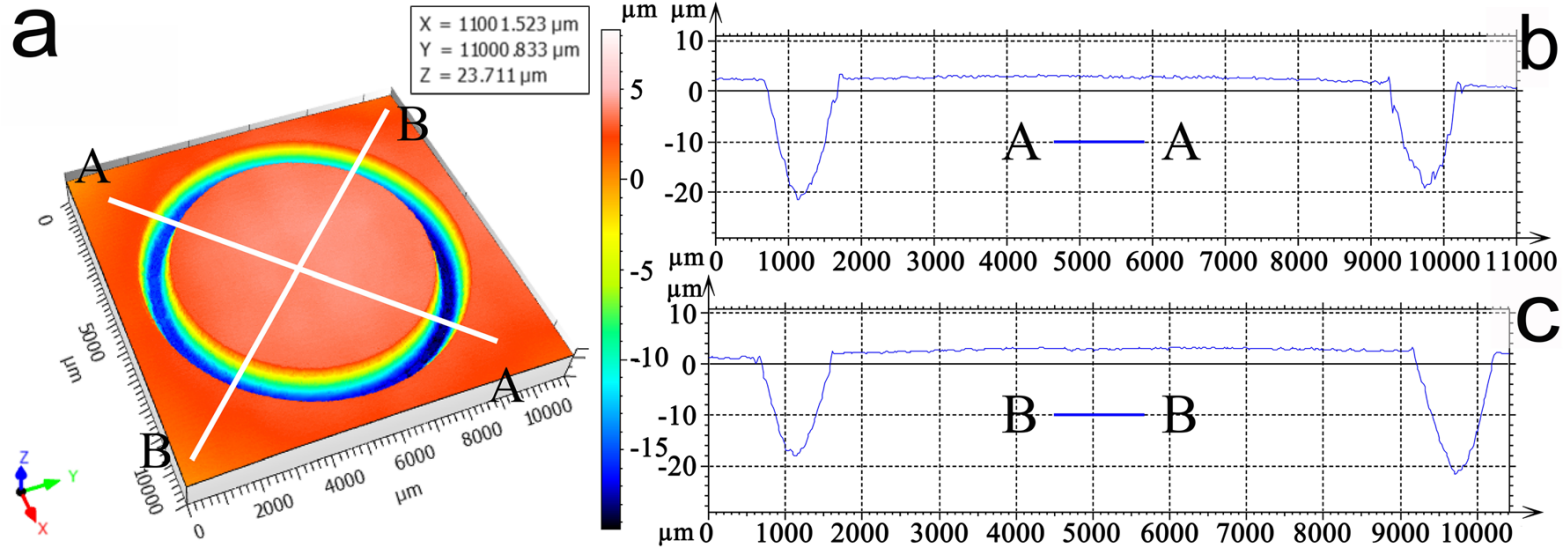
(a) Typical 3D pattern of the wear profile, and (b) and (c) 2D wear profiles extracted from the straight lines AA and BB of Fig. 5b.

Following ASTM Standard No. G99-95,^20,21^ the frictional coefficients were recorded in the computer-controlled system at a speed to 0.25 m s^−1^, during 80 min and 95 min wear. After three tests, the wear rates *W* could be calculated by the equation: *W* = (*A*·*C*)/(*P*·*S*),^15,22^ where the parameters, *S*, *P*, *C*, and *A* are the sliding distance, applied load, friction perimeter, and cross-sectional area, respectively. The typical morphology of an overall wear scar is shown in Fig. 4b; the amplified EPMA morphology of the wear scar of the rectangle region in Fig. 4b is shown in Fig. 4c. Subsequently, 2D wear profiles were continuously extracted from the lines AA and BB of Fig. 5a for evaluating the cross-sectional area *A*, and the results are correspondingly indicated in Fig. 5b and c, respectively. All the tests were carried out three times to ensure a mean value for the cross-sectional area *A*. Subsequently, the wear rates *W* could be calculated with the beneficial assistance of the cross-sectional area.

## 3. Results and discussion

### 3.1. Analysis of the tribological properties and regulation functions

**Fig. 6:**
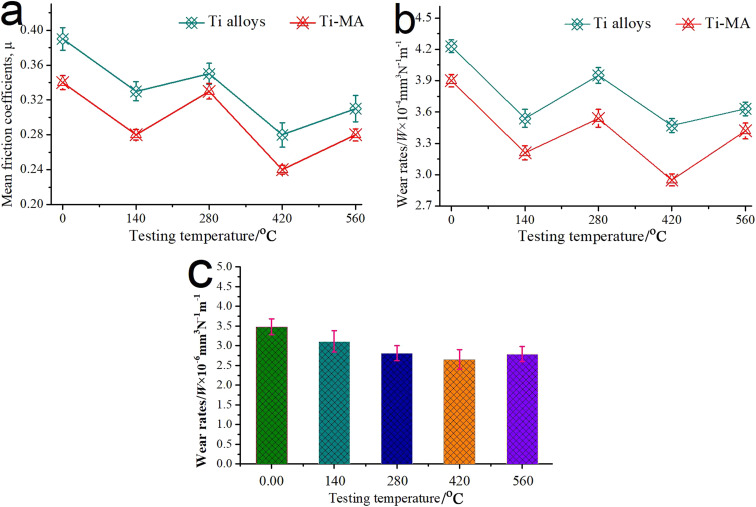
(a) Typical friction coefficients and (b) wear rates for 80 min under different temperatures of the Ti alloys and Ti-MA samples; (c) mean wear rates of spherical Si_3_N_4_ sliding against the Ti-MA.

Fig. 6 shows the typical friction coefficients and wear rates for 80 min of the LAM samples under different temperatures at the load of 12 N. As shown in Fig. 6, MA was added into the Ti-based alloys and showed excellent regulation functions for the good lubrication of MA, resulting in the better tribological behaviors of Ti-MA over that of the pure Ti-alloy, at temperatures from 0 °C to 560 °C. In particular, significant attention should be paid to achieving the best friction and wear behaviors of pure Ti-alloys and for Ti-MA at 420 °C. Fig. 6c shows the mean wear rates of spherical Si_3_N_4_ sliding against Ti-MA, under temperatures ranging from 0 °C to 560 °C. As shown in Fig. 6c, the mean wear rate of the Si_3_N_4_ ball was observed to be smaller at 420 °C compared with those at other temperatures. From the aforementioned discussions, the friction coefficients and wear rates at 420 °C of the Ti-MA sample and its matched pair (spherical Si_3_N_4_) were smaller than those at other temperatures.

**Fig. 7:**
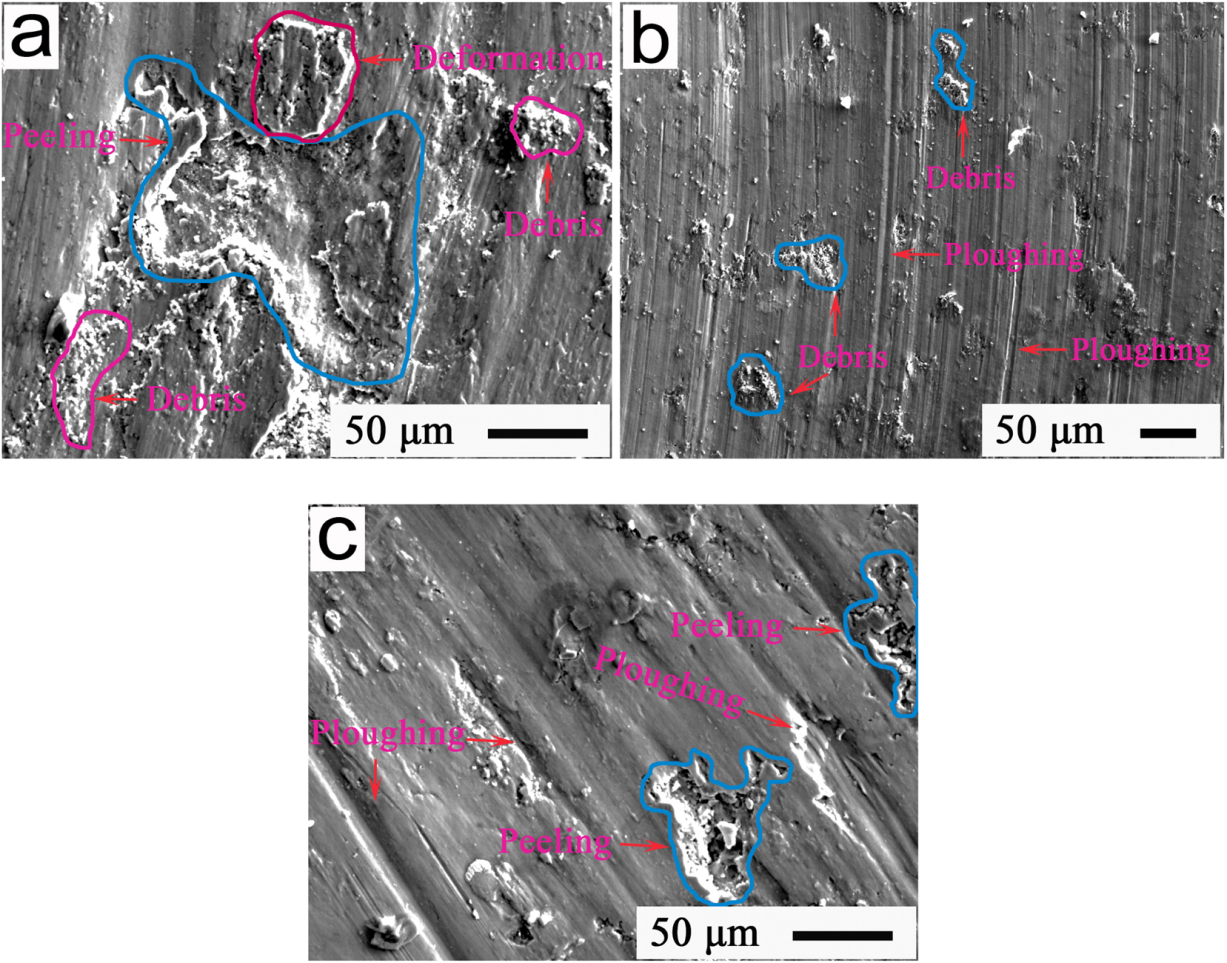
EPMA patterns indicating the typical wear morphologies after 80 min for friction interfaces of pure Ti-alloys at (a) 280 °C, (b) 420 °C, and (c) 560 °C.

Further, the tribological properties of Ti-matrix alloys should be characterized using EPMA to provide an effective way to achieve their tribological control. Fig. 7 represents the EPMA patterns, indicating the typical wear morphologies at 12 N of the friction interfaces of Ti alloys under different temperatures. As shown in Fig. 7a, massive wear debris, conspicuous deformation, and a large peeling pit could be observed from the wear interfaces; indicating that severe peeling was liable to occur at 280 °C due to the heavy wear and high friction resistance, which was harmful for interface lubrication, resulting in a friction coefficient of approximately 0.35 and wear rate of approximately 3.95 × 10^−4^ mm^3^ N^−1^ m^−1^. As shown in Fig. 7b, due to the inconspicuous plowing and small quantity of wear debris, the main wear mechanism was a slight plowing of the Ti alloys at 420 °C, with a friction coefficient of about 0.28 and a wear rate of approximately 3.47 × 10^−4^ mm^3^ N^−1^ m^−1^. Looking at Fig. 7c, it could be seen that the plowing and peeling were regarded as the primary wear mechanisms at 560 °C, which induced a friction coefficient and wear rate of ∼0.31 and 3.63 × 10^−4^ mm^3^ N^−1^ m^−1^, respectively.

**Fig. 8:**
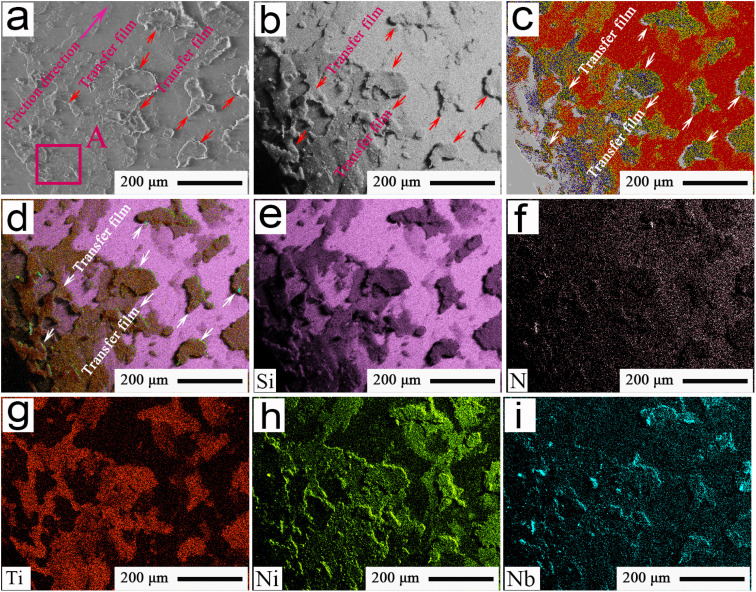
Typical FESEM morphology (a), backscattered electron image (b), mapping patterns (c) and (d), and main element distributions (e)–(i) of the friction interface at 420 °C of the spherical Si_3_N_4_ sliding against the Ti alloys.

After 80 min sliding, Fig. 8a shows the FESEM pattern, showing the typical morphology at 12 N of a friction interface of spherical Si_3_N_4_. The EDS results showed that almost 8.12 wt% Ti and 2.03 wt% Ni were found in the red rectangle A area in Fig. 8a. A backscattered electron (BSE) image of Fig. 8a is shown in Fig. 8b. The corresponding mapping patterns of Fig. 8a are indicated in Fig. 8c and d. The main element distributions of material in Fig. 8a can be seen from Fig. 8e–i. As shown in Fig. 8e and f, the presence of the elements Si and N ensured that the main components of the substrates of Si_3_N_4_ balls could be diagnosed. The main distributions of the elements, Ti, Ni, and Nb were found on a friction interface of the Si_3_N_4_, as shown in Fig. 8g–i. This verified that the Ti alloy had been transferred to the spherical Si_3_N_4_, and formed the observed films, as can be seen from Fig. 8a–d. It can be deduced from these figures that during wear, the Ti alloys transferred to the friction surface of the Si_3_N_4_ ball, and produced deformation under the action of frictional force. This facilitated a significant flowing along the sliding direction of the wear, which resulted in the conspicuous improvement of the friction and wear performance at 420 °C, compared to those at other temperatures.

**Fig. 9:**
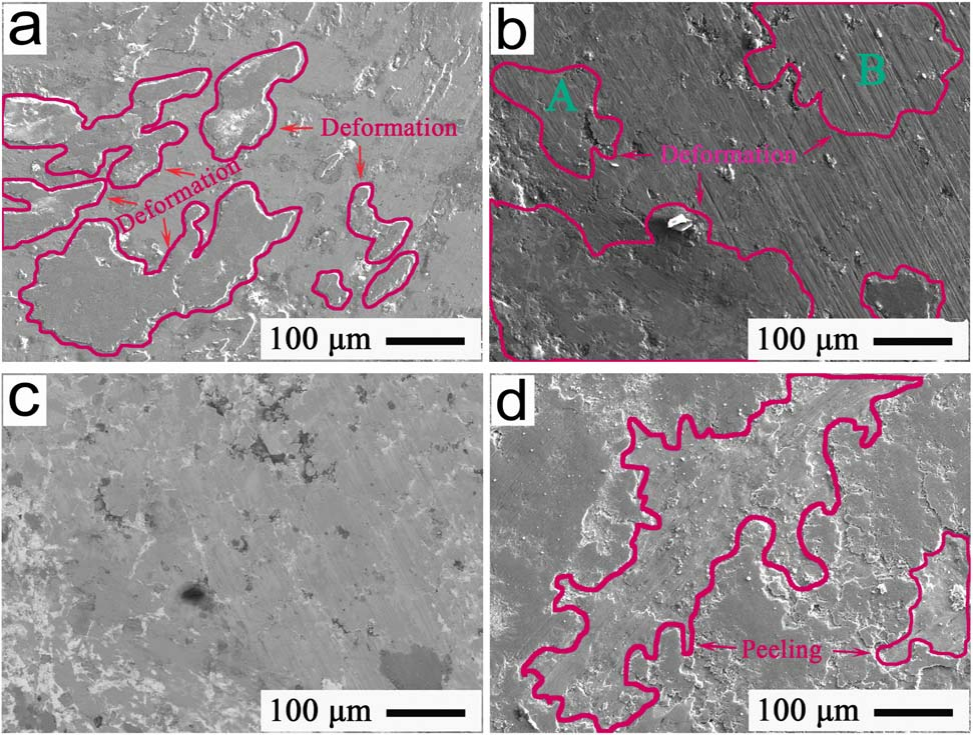
EPMA images showing the typical morphologies for 80 min for the friction interfaces at 12 N of Ti-MA at (a) 280 °C, (b) and (c) 420 °C, and (d) 560 °C.

To provide an effective way for achieving MA regulation, the EPMA images in Fig. 9 showed the typical interface morphologies of the sliding wear of Ti-MA at 280 °C, 420 °C, and 560 °C. As shown in Fig. 9a, a significant number of regions with island deformations were found on the frictional interface. This indicated that the MA alloys at 280 °C continuously migrated from the microchannels to a frictional interface, leading to their inhomogeneous enrichment and the formation of island deformation, thus acquiring a friction coefficient of about 0.31 and a wear rate of almost 3.63 × 10^−4^ mm^3^ N^−1^ m^−1^. Fig. 9b shows the typical EPMA morphologies at 420 °C of the friction interface of Ti-MA, while the BSE image of Fig. 9b is indicated in Fig. 9c. As shown in Fig. 9b and c, a big region of plastic deformation could be observed on the smooth friction interface. This indicated the migration from a microchannel of the MA, leading to a sufficient enrichment on the friction interface appearing during the wear. Subsequently, the MA alloys were well spread out to induce the formation of MA-enriched deformation regions. The MA at 420 °C produced the excellent plasticity deformation to repair the damages of the frictional interface. Such a temperature (420 °C) can be approximated to the melting temperature (almost 450 °C) of MA,^14^ allowing the interface to exhibit a good deformation to execute a hopeful reparation, and performing a key role in producing effective self-healing to acquire a smooth morphology of the wear interface. Because of this, the MA regulation functions were significantly enhanced, which resulted in a smaller friction coefficient and wear rate of approximately 0.24 and 2.95 × 10^−4^ mm^3^ N^−1^ m^−1^, respectively, compared with those at 280 °C.

As shown in Fig. 9d, large peeling regions were formed on the friction interface at 520 °C, which was higher than the MA melting temperature (approximately 450 °C). This excessive temperature rendered the MA alloys to be melted, and thus it was observed that a significant amount of MA alloys flowed out of the frictional interface because of the high centrifugal force of the sliding wear. This left only a small quantity of MA lubricant on the friction interface, which made it difficult to effectively repair the frictional interface, resulting in big peeling regions. This meant a poor self-healing function of the MA, which in turn caused the Ti-MA to exhibit an insufficient regulation. Consequently, a friction coefficient of approximately 0.28 and wear rate of approximately 3.42 × 10^−4^ mm^3^ N^−1^ m^−1^ at 520 °C were obtained, which were higher than those at 420 °C. The aforementioned discussions show that the best friction and wear behavior of Ti-MA appeared at 420 °C, among the chosen and tested temperatures.

**Fig. 10:**
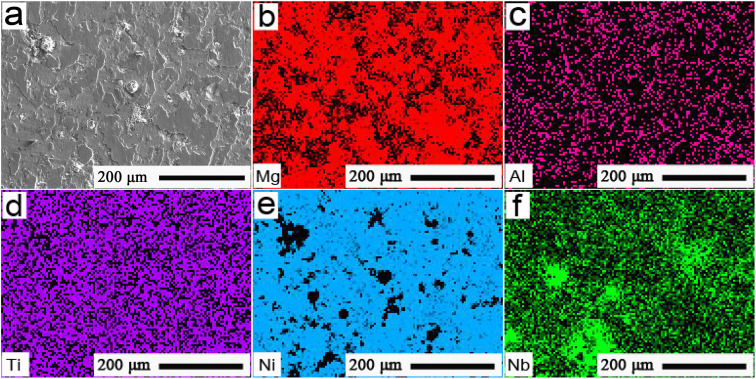
(a) EPMA images showing the typical wear morphology and (b)–(f) main element distributions at 280 °C and 12 N of the friction interfaces.

Fig. 10 shows the EPMA patterns at 280 °C that present the typical wear morphologies and main element distributions of the friction interfaces for 80 min. As shown in Fig. 10a–c, the elements Mg and Al were observed as mainly appearing in the regions of island deformation. During wear, the MA alloys experienced plastic deformation at 280 °C and formed massive regions showing island deformation. Meanwhile, the main elements, Ti, Ni, and Nb, were also found on the friction interface, as shown in Fig. 10d–f. From the discussions about Fig. 9a, the MA migrated from the microchannel to produce an inhomogeneous enrichment on the friction interface, leading to the island deformation at 280 °C.

**Fig. 11:**
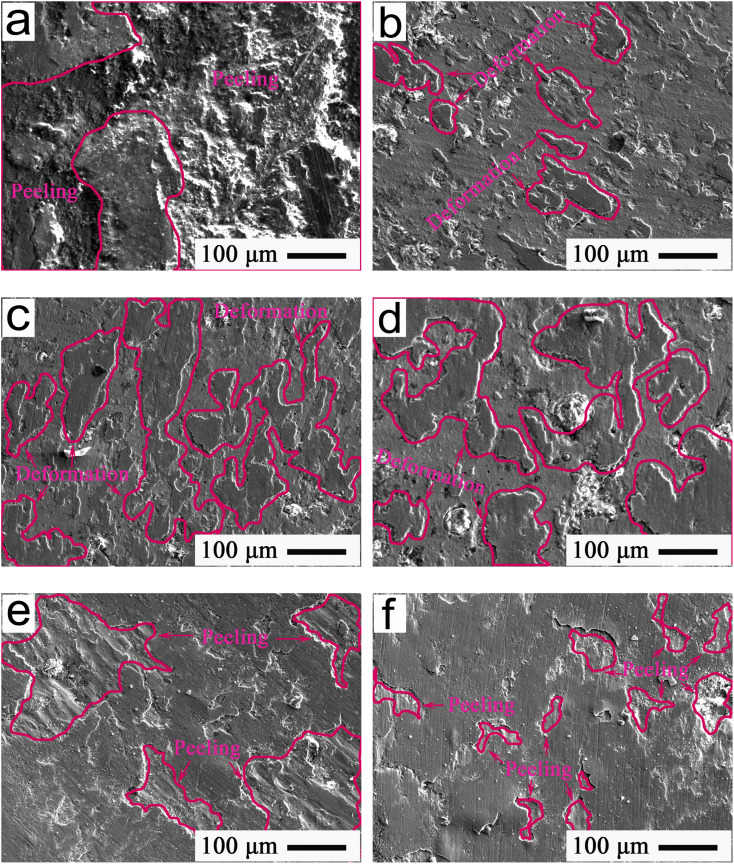
FESEM images showing the typical morphologies of the friction interfaces at 420 °C and 24 N with the increase in sliding time from 80 min to 95 min.

To study the continuous influence of the self-regulating and self-healing functions on the friction and wear behaviors of the Ti-MA at 420 °C, the sliding time was extended up to 95 min from 80 min, and the applied loads increased up 24 N from 12 N. The mean friction coefficient (∼0.31) and wear rate (about 4.15 × 10^−4^ mm^3^ N^−1^ m^−1^) from 77 min to 80 min were obtained, which were used as the benchmark to analyze the influence of the interface healing on the tribological properties. An FESEM image of the typical wear morphology at 80 min of the Ti-MA interface is presented in Fig. 11a. As shown in Fig. 11a, large peeling pits appeared in the friction interface at 80 min. When the sliding wear test was carried out for up 83 min from 80 min, the corresponding FESEM image showing the representative morphology of a friction interface is shown in Fig. 11b. As shown in Fig. 11b, at 83 min, micro-regions of island deformation were found to appear on the friction interface. As the friction and wear tests continued from 86 min to 89 min, the island micro-regions started to grow in succession to form larger deformation regions, which can be seen in Fig. 11c at 86 min and Fig. 11d at 89 min. Because of this, the MA alloys exhibited enhanced regulation functions, showing a reduced friction coefficient and wear rate of almost 0.28 and 3.47 × 10^−4^ mm^3^ N^−1^ m^−1^, respectively. With an unremitting increase from 92 min to 95 min of sliding time, the number of deformation regions reduced, but the deformation area of friction interface of the Ti-MA increased, which can be seen from Fig. 11d at 89 min, Fig. 11e at 92 min, and Fig. 11f at 95 min. This allowed the frictional interface to be continually repaired to form the self-healing morphology, and caused the peeling area to be continuously reduced and leading to the formation of a lot of small pits. These results were consistent with the published report in ref. 14. Hence, the regulation functions of the MA alloys were increasingly enhanced, which rendered the friction coefficient and wear rate to be further reduced to approximately 0.25 and 2.98 × 10^−4^ mm^3^ N^−1^ m^−1^, respectively.

### 3.2. Enhancement functions of the tribological regulation

**Fig. 12:**
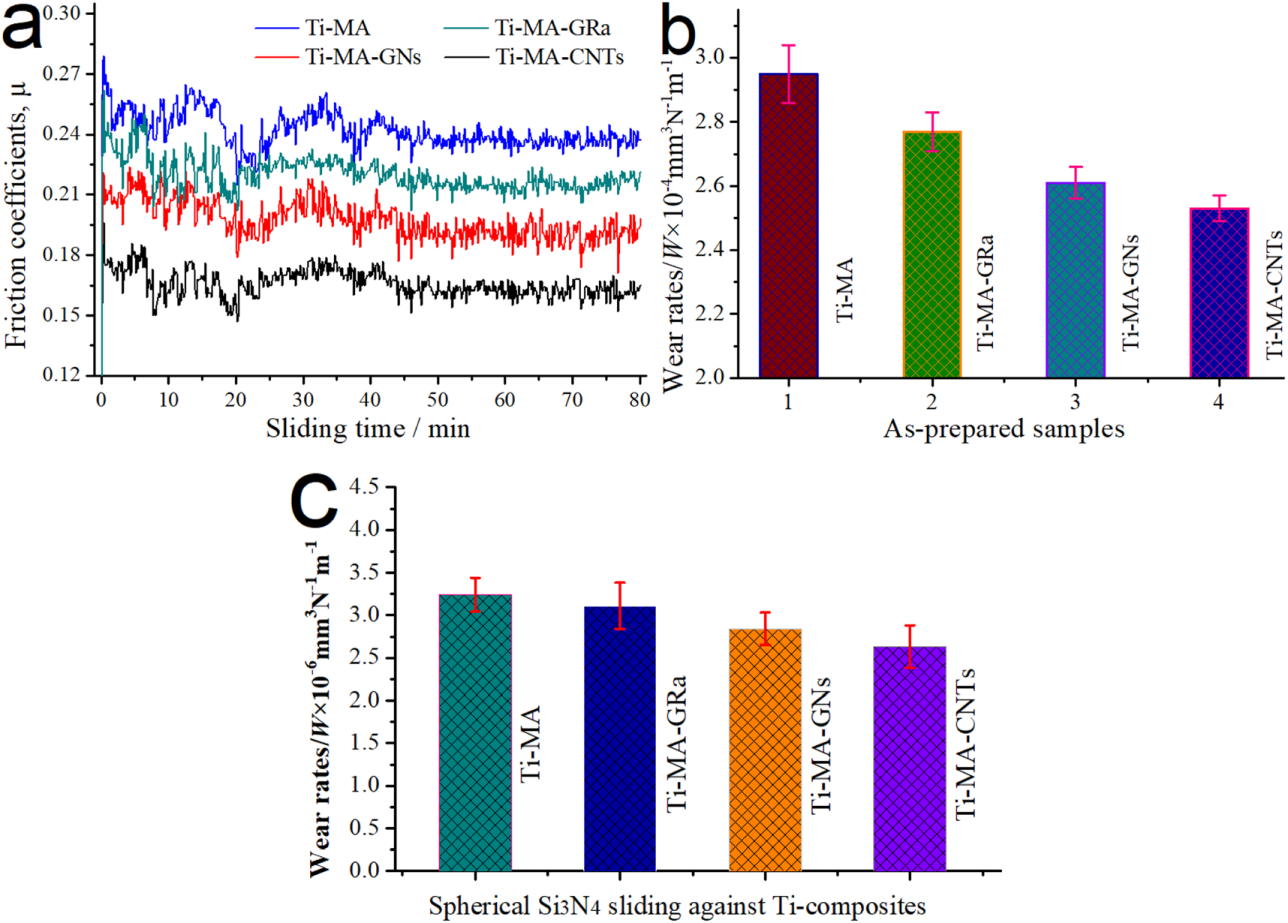
(a) Typical friction coefficients and (b) wear rates at 420 °C and 12 N for Ti-MA, Ti-MA-GRa, Ti-MA-GNs, and Ti-MA-CNTs; (c) mean wear rates of the spherical Si_3_N_4_.

In order to study the respective collaboration functions of MA-GRa, MA-GNs, and MA-CNTs on the tribological regulation of Ti-MA, the typical friction coefficients and wear rates for 80 min for Ti-MA, Ti-MA-GRa, Ti-MA-GNs, and Ti-MA-CNTs are exhibited in Fig. 12. As shown in Fig. 12a and b, the tribological behaviors at 420 °C of Ti-MA-GRa, Ti-MA-GNs, and Ti-MA-CNTs were superior to those of Ti-MA. This showed that the MA respectively cooperated with the GRa, GN, and CNTs for improving the functional regulation performance, effectively leading to small coefficients for the friction and wear rates. Furthermore, Ti-MA-GRa showed poor tribological behaviors, as compared to those of Ti-MA-GNs, while the friction and wear behavior of Ti-MA-CNTs was superior. It was revealed that significant differences in the cooperative functions for MA-GRa, MA-GNs, and MA-CNTs were found to regulate the friction and wear behavior. Fig. 12c shows mean wear rates of the spherical Si_3_N_4_. As shown in Fig. 12c, the mean wear rate of spherical Si_3_N_4_ sliding against Ti-MA-CNTs was observed to be smaller, compared with those of the Si_3_N_4_ balls that were slid against the other Ti-based composites.

**Fig. 13:**
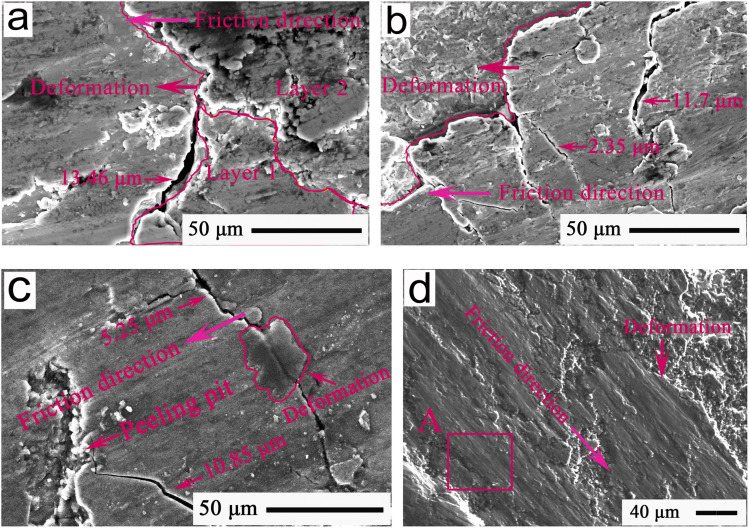
FESEM images showing the typical morphologies for 80 min of friction interfaces at 420 °C and 12 N of (a) Ti-MA-GRa, (b) Ti-MA-GNs, and (c) Ti-MA-CNTs; (d) interface morphology of spherical Si_3_N_4_ sliding against the Ti-MA-CNTs.

To explore the regulation functions of MA-GRa, MA-GNs, and MA-CNTs, microcosmic images showing the typical morphologies of frictional interfaces after 80 min sliding for the Ti-MA-GRa, Ti-MA-GNs, and Ti-MA-CNTs are indicated in Fig. 13. The surface crack widths and self-healing ratios of Ti-MA-GRa, Ti-MA-GNs, and Ti-MA-CNTs, compared to the Ti-MA, are indicated in Table 1. As shown in Fig. 13a and this table, the maximum and minimum widths of the cracks were 13.46 and 7.21 μm, respectively, while the mean width was calculated to be approximately 11.34 μm. In the deformation regions, the obvious flowing led to layer 2 overlapping layer 1. Subsequently, the MA-GRa lubricants were filled into the surface cracks, which caused the crack width to be reduced down to approximately 7.21 μm, resulting in a hopeful repair of the friction interface, with that of Ti-MA-GRa being significantly smaller than that (almost 35.72 μm) of Ti-MA. According to ref. 20, the lubricant Ti_3_SiC_2_ can be used to increase the plastic flowing of SnAgCu, and could allow an efficient filling of the surface cracks of the SnAgCu, thus accelerating interfacial healing to self-regulate the friction and wear behavior. Hence, it could be deduced that an interlayer separation of graphite would accelerate the plastic flowing of MA, and improve the interface repairment function of Ti-MA-GRa, thereby resulting in the width of the surface cracks being smaller than that of the Ti-MA. Here, the self-healing ratio *η* of the Ti-MA-GRa was almost 68.25%, as calculated from the equation *η* = (CW_Ti-MA_-CW)/CW_Ti-MA_, where CW_Ti-MA_ is the mean width of Ti-MA cracks and CW is the mean width of surface cracks for Ti-MA-GRa, Ti-MA-GNs, or Ti-MA-CNTs. Hence, the self-healing ratio proved the good self-recovery functions of MA-GRa, and showed the good regulation behaviors during wear, resulting in a friction coefficient of almost 0.22, along with a wear rate of about 2.76 × 10^−4^ mm^3^ N^−1^ m^−1^ (see Fig. 12).

**Table 1:** Surface crack width and self-healing ratio of Ti-MA-GRa, Ti-MA-GNs, and Ti-MA-CNTs relative to Ti-MA

LAM samples	Surface crack width (μm)					
Ti-MA-GRa	Maximum	13.46	Minimum	7.21	Mean width	11.34
	Self-healing ratio				68.25%	
Ti-MA-GNs	Maximum	11.70	Minimum	2.35	Mean width	10.05
	Self-healing ratio				71.86%	
Ti-MA-CNTs	Maximum	10.85	Minimum	5.25	Mean width	8.39
	Self-healing ratio				76.51%	

As shown in Fig. 13b and Table 1, excellent flowability was observed in the deformation region, which led to an increase in the deformation areas, which further resulted in an enhancement of the self-healing function. Ti-MA-GNs displayed a crack width of 10.05 μm, which was smaller than that of Ti-MA-GRa; further, the self-healing ratio (almost 71.86%) was higher compared to that of the Ti-MA-GRa. It was possible that when compared with GRa, the graphene nanosheets (GNs) were thinner, which made it easier for them to slide, thus producing the more plastic deformation of MA. This was then helpful for the self-healing of the surface cracks, thus enhancing the regulating behaviors. Ti-MA-GNs showed a friction coefficient of approximately 0.20 and wear rate of about 2.58 × 10^−4^ mm^3^ N^−1^ m^−1^, as can be seen from Fig. 12, both smaller than those of Ti-MA-GRa.

As can be seen from Fig. 13c and Table 1, crack widths ranging from 5.25 μm to 10.85 μm were found in the friction interfaces. The mean crack width was calculated as 8.39 μm. A deformation region existed on the cracks to effectively recover the friction interface. Hence, Ti-MA-CNTs obtained a self-healing ratio of approximately 76.51%, which was higher than those of Ti-MA-GRa and Ti-MA-GNs. The excellent self-healing function led to the observed enhancements of the regulating behaviors of Ti-MA-CNTs, along with a low friction coefficient of almost 0.17 and wear rate of about 2.51 × 10^−4^ mm^3^ N^−1^ m^−1^, which were smaller than those of Ti-MA-GRa and Ti-MA-GNs (see Fig. 12).

A typical pattern showing the FESEM interface morphology of spherical Si_3_N_4_ sliding against the Ti-MA-CNTs is shown in Fig. 13d. As shown in this figure, the plastic deformation materials were abundantly enriched on the friction interface, and formed a large area of plastic deformation. The EDS results of the red rectangle A in Fig. 13d were approximately 9.74 wt% Al, 6.42 wt% Mg, 0.59 wt% C, 3.74 wt% Ti, and 1.12 wt% Ni. During wear, the MA, CNTs, and Ti alloys from the Ti-MA-CNTs were transferred to the Si_3_N_4_ ball, thus leading to the observed deformation under the sliding friction force, which was helpful for reduction of the friction resistance and material loss. This allowed the superior tribological behaviors of Ti-MA-CNTs compared to those of Ti-MA-GRa and Ti-MA-GNs.

**Fig. 14:**
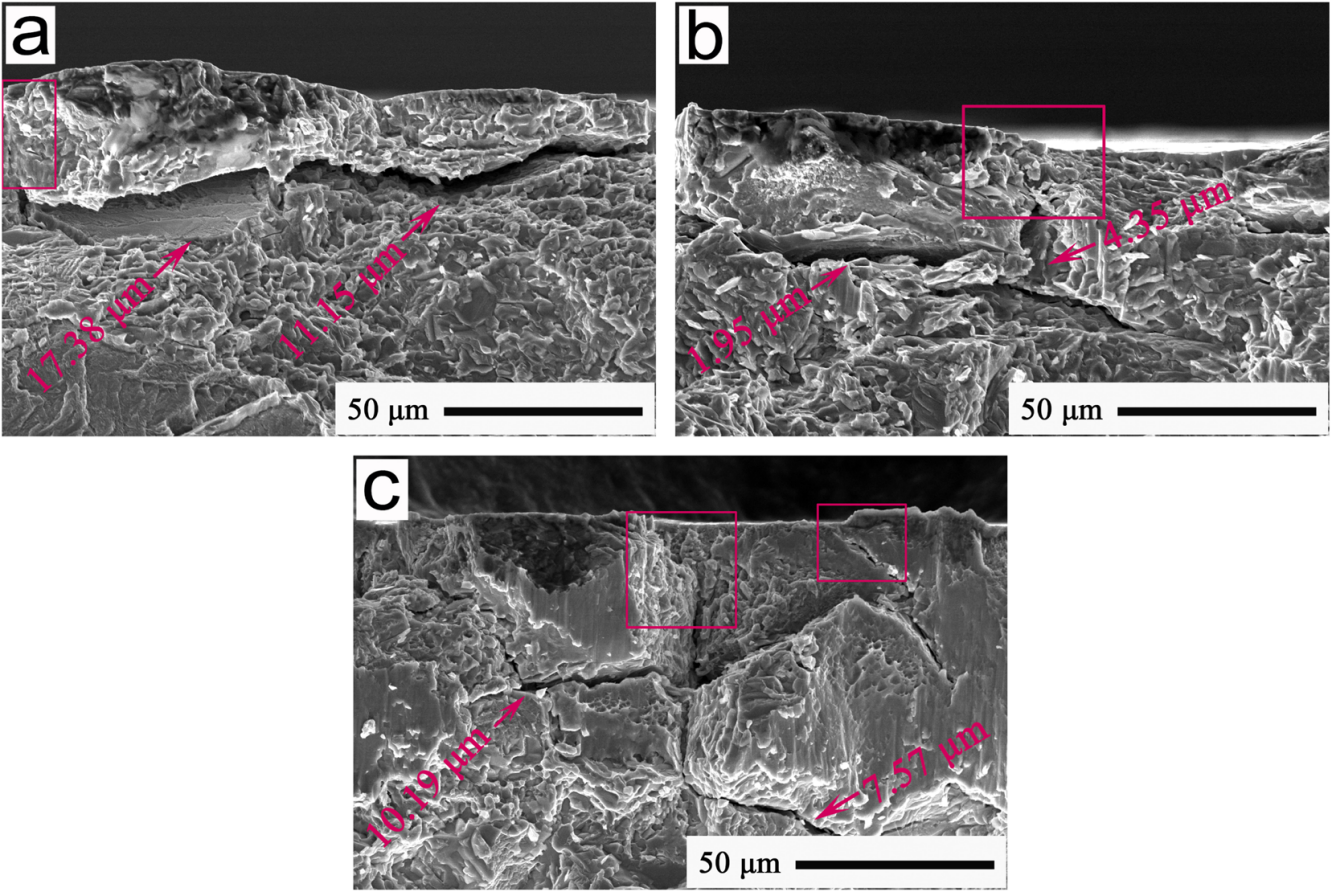
FESEM cross-section images at 12 N showing the typical wear morphologies at 420 °C of (a) Ti-MA-GRa, (b) Ti-MA-GNs, and (c) Ti-MA-CNTs.

Fig. 14 shows a typical FESEM cross-section image at an applied load of 12 N for the wear morphologies of Ti-MA-GRa, Ti-MA-GNs, and Ti-MA-CNTs. Table 2 shows the cross-sectional crack width and the crack self-healing ratio relative to the Ti-MA samples. As can be seen from Fig. 14 and Table 2, the cross-section cracks were observed to be larger than the surface cracks that can be seen in Fig. 13 and Table 1. This indicated that during wear, cracks were produced in the cross-sections, and gradually extended to the friction interface, and then increased the crack growth. Microscopic cracks of the friction interfaces could be self-recovered, and the healing areas are indicated in the red rectangles in Fig. 14. This verified that the cracks self-recovered first in the top cross-section, gradually expanding down to the cross-section bottom, to finally show a self-healing morphology during wear. This rendered the regulation functions of MA-GRa, MA-GNs, and MA-CNTs as excellent, inducing Ti-MA-GRa, Ti-MA-GNs, and Ti-MA-CNTs to achieve excellent lubrication performances compared to Ti-MA.

**Table 2:** Cross-sectional crack width and crack self-healing ratio relative to the Ti-MA samples of Ti-MA-GRa, Ti-MA-GNs, and Ti-MA-CNTs

LAM samples	Cross-sectional crack width (μm)					
Ti-MA-GRa	17.38	14.86	16.52	15.74	12.39	11.15
	Mean crack width		14.67	Self-healing ratio		46.28%
Ti-MA-GNs	13.35	10.95	11.48	13.07	12.32	8.95
	Mean crack width		11.69	Self-healing ratio		57.20%
Ti-MA-CNTs	10.19	9.67	8.53	9.83	9.21	7.57
	Mean crack width		9.16	Self-healing ratio		66.46%

Compared to those of Ti-MA-GRa and Ti-MA-GNs, smaller widths of the cross-section cracks of Ti-MA-CNTs were observed. This ensured that the CNTs were well coated using MA to form the MA-CNTs, allowing the CNTs to produce a rolling friction. This could accelerate the plastic flow of MA, which is conducive for helping the healing of the friction interface, thus forming a smooth interface. During wear, this interface of the Ti-MA-CNTs helped significantly reduce the sliding friction resistance and the surface material loss, thus resulting in a smaller friction coefficient and wear rate of the Ti-MA-CNTs than those of Ti-MA-GRa and Ti-MA-GNs.

**Fig. 15:**
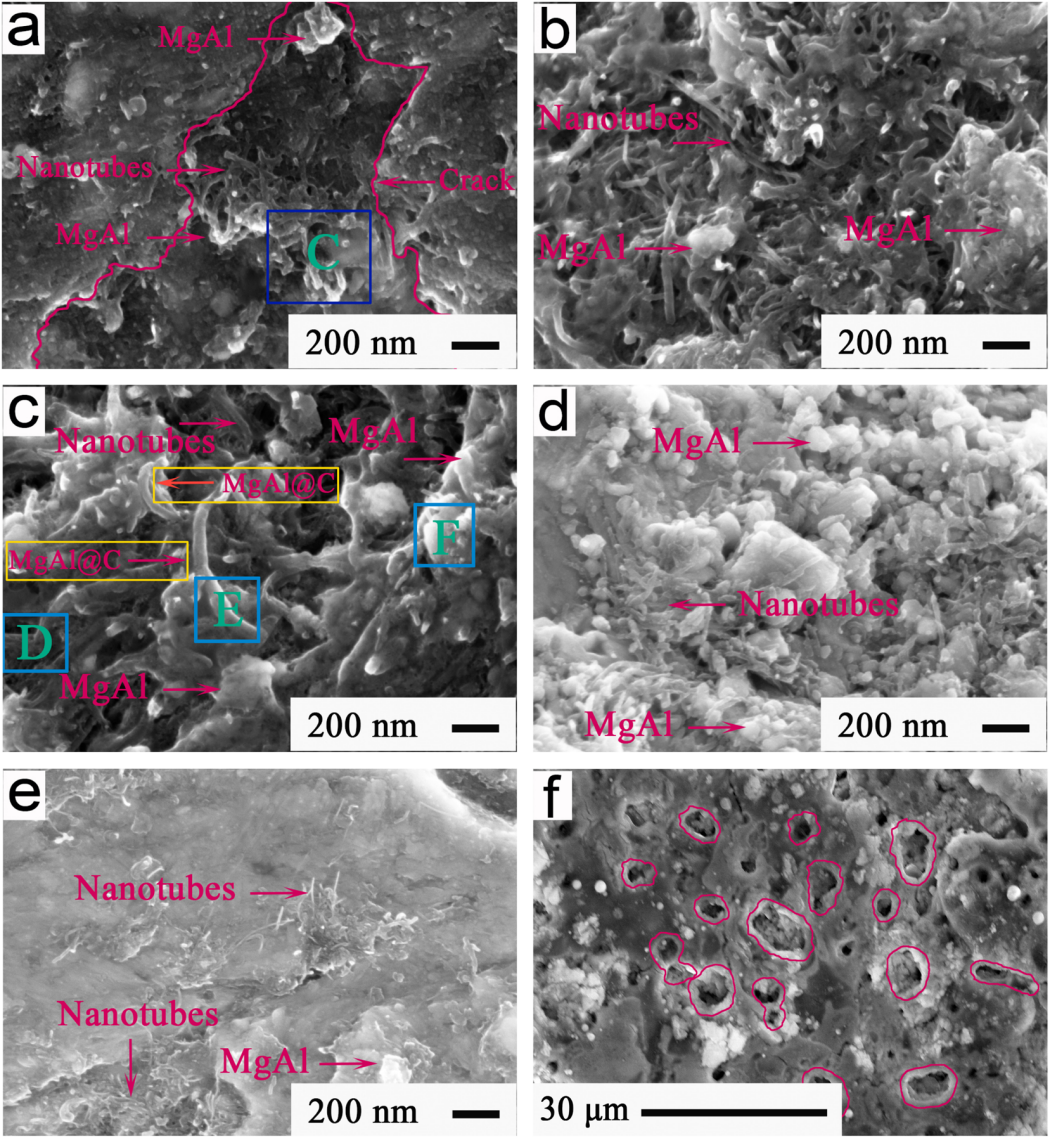
FESEM patterns showing (a)–(e) the typical morphologies from 80 min to 95 min, and (f) cross-section structure of the friction interface at 420 °C and 12 N of Ti-MA-CNTs.

Fig. 15 shows the FESEM patterns indicating the typical morphologies from 80 min to 95 min of the friction interfaces at 420 °C and 12 N for Ti-MA-CNTs. As shown in Fig. 15, during the friction and wear process, a large amount of CNTs were gradually exposed to the friction interface at 83 min, as shown in Fig. 15a. The contents (wt%) of the main elements in the rectangle C in Fig. 15a were 4.07-C, 20.13-Mg, 23.72-Al, and 6.41-O. As can be seen from Fig. 15b, the CNTs were observed to be well combined with the MA at 86 min, and accelerated MA enrichment on the friction interface. As shown in Fig. 15c, the main element contents of the rectangles D, E, and F in Fig. 15c were measured. The EDS results of rectangle D were 21.04 wt% Mg, 24.12 wt% Al, and 4.97 wt% C. Similarly, 20.74 wt% Mg, 23.92 wt% Al, and 4.73 wt% C were found in rectangle E. A similar composition was observed in rectangle F, namely: 23.24 wt% Mg, 29.75 wt% Al, and 4.12 wt% C. These results indicated that, at 89 min, the CNTs had been coated using a massive amount of MA alloys (MgAl@C) to form the deformation regions.

As shown in Fig. 15d, the MA-CNTs showed an excellent coordination function to abundantly gather on the friction interface at 92 min, and further formed an excellent connection. When the sliding wear test was carried out to 95 min, the CNTs were combined continually with the MA, and produced a helpful spread under the sliding force, thus forming a smooth friction interface, as can be seen from Fig. 15e. Fig. 15f shows a typical cross-section structure of a friction interface. As shown in Fig. 15f, massive micropores appeared in the microchannels, indicating that the MA-CNTs had abundantly migrated from the channels to well enrich the friction interface at 95 min. During wear, the MA-CNTs were observed to have an excellent deformation flowing, and then spread out uniformly to repair the surface cracks, thereby leading to the formation of a smooth morphology. The self-healing functions of the frictional interfaces were improved, along with the regulation behavior of the MA-CNTs, which led to a small friction coefficient and a lowered wear rate. Consequently, the Ti-MA-CNTs exhibited better friction and wear performances than those of Ti-MA-GRa and Ti-MA-GNs.

## 4. Conclusions

For extending the high-temperature applications of gear-driven systems, the tribological behaviors and regulating functions of the microchannel interfaces are a key focus of research. The following conclusions could be derived from this study of these aspects:

(1) A service temperature of 420 °C near the melting temperature (almost 450 °C) of MA allowed the MA to show good deformation, and improved the self-healing and regulation functions. This allowed Ti-MA to obtain a smaller friction coefficient and wear rate compared to those of other Ti-based alloys.

(2) MA-GRa, MA-GNs, and MA-CNTs showed superior cooperation functions, which meant they could effectively regulate a smaller friction coefficient and wear rate compared to the individual MA alloy.

(3) The interlayer separation of GRa accelerated the plastic flowing of MA, and improved the interface repairment and self-healing function of Ti-MA-GRa, thereby regulating the friction resistance and material loss. This resulted in a smaller friction coefficient and wear rate of Ti-MA-GRa compared to that of Ti-MA.

(4) When compared with GRa, the graphene nanosheets were easier to slide, and produced a better deformation of MA, facilitating a good self-healing of the surface cracks. This enhanced the regulating behaviors of Ti-MA-GNs, and resulted in a small friction coefficient of about 0.20 and wear rate of approximately 2.58 × 10^−4^ mm^3^ N^−1^ m^−1^.

(5) During wear, the CNTs made the MA become significantly enriched, showing a good combination to carry out the collaborative functions, which could effectively repair the surface cracks to improve the self-healing of the wear interface. Hence, the tribological performances of Ti-MA-CNTs were well regulated, demonstrating a friction coefficient of almost 0.17 and a wear rate of approximately 2.51 × 10^−4^ mm^3^ N^−1^ m^−1^, which were smaller compared with those of Ti-MA-GRa and Ti-MA-GNs. This would also provide vital space for significantly promoting the high-temperature applications of gear-driven systems.

## Conflicts of interest

There are no conflicts to declare.

## Supplementary Material
